# Supplementary material for: CelEst: a unified gene regulatory network for estimating transcription factor activities in C. elegans
Source: Genetics. 2024 Dec 20;229(3):iyae189. doi: 10.1093/genetics/iyae189 (PMC11912867; doi:10.1093/genetics/iyae189)
Supplement: iyae189_Supplementary_Data [file iyae189_supplementary_data.zip › Figure_S1_GENETICS-2024-307499.pdf]

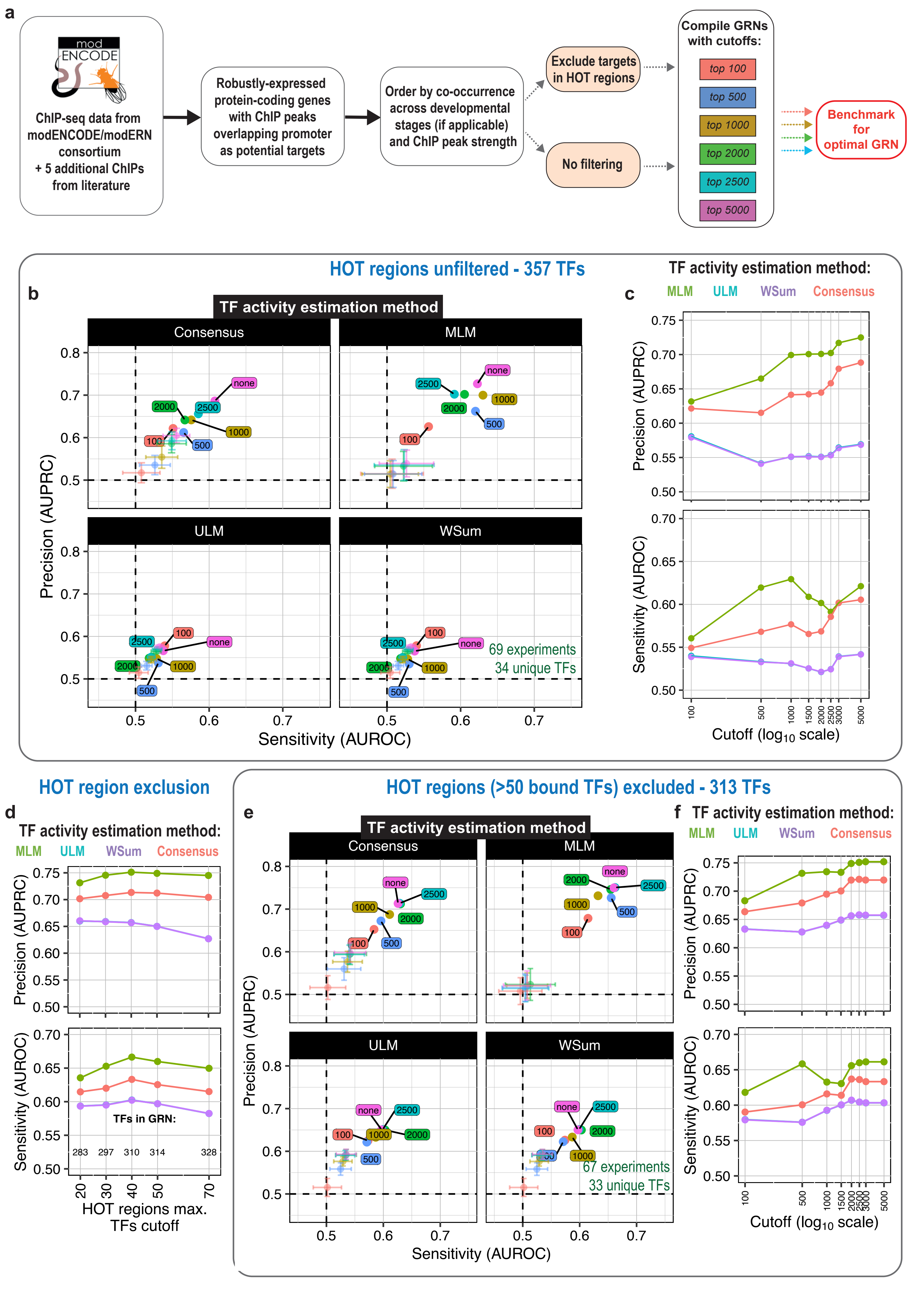
a
mod ENCODE
ChIP-seq data from modENCODE/modERN consortium + 5 additional ChIPs from literature
Robustly-expressed protein-coding genes with ChIP peaks overlapping promoter as potential targets
Order by co-occurrence across developmental stages (if applicable) and ChIP peak strength
Exclude targets in HOT regions
No filtering
Compile GRNs with cutoffs:
top 100
top 500
top 1000
top 2000
top 2500
top 5000
Benchmark for optimal GRN
HOT regions unfiltered - 357 TFs
b
TF activity estimation method
Consensus
MLM
ULM
WSum
Precision (AUPRC)
Sensitivity (AUROC)
69 experiments
34 unique TFs
c
TF activity estimation method:
MLM ULM WSum Consensus
Cutoff (log_{10} scale)
HOT region exclusion
d
HOT regions max. TFs cutoff
TFs in GRN:
283 297 310 314 328
HOT regions (>50 bound TFs) excluded - 313 TFs
e
67 experiments
33 unique TFs
f
