# Supplementary material for: CelEst: a unified gene regulatory network for estimating transcription factor activities in C. elegans
Source: Genetics. 2024 Dec 20;229(3):iyae189. doi: 10.1093/genetics/iyae189 (PMC11912867; doi:10.1093/genetics/iyae189)
Supplement: iyae189_Supplementary_Data [file iyae189_supplementary_data.zip › Figure_S8_GENETICS-2024-307499.pdf]

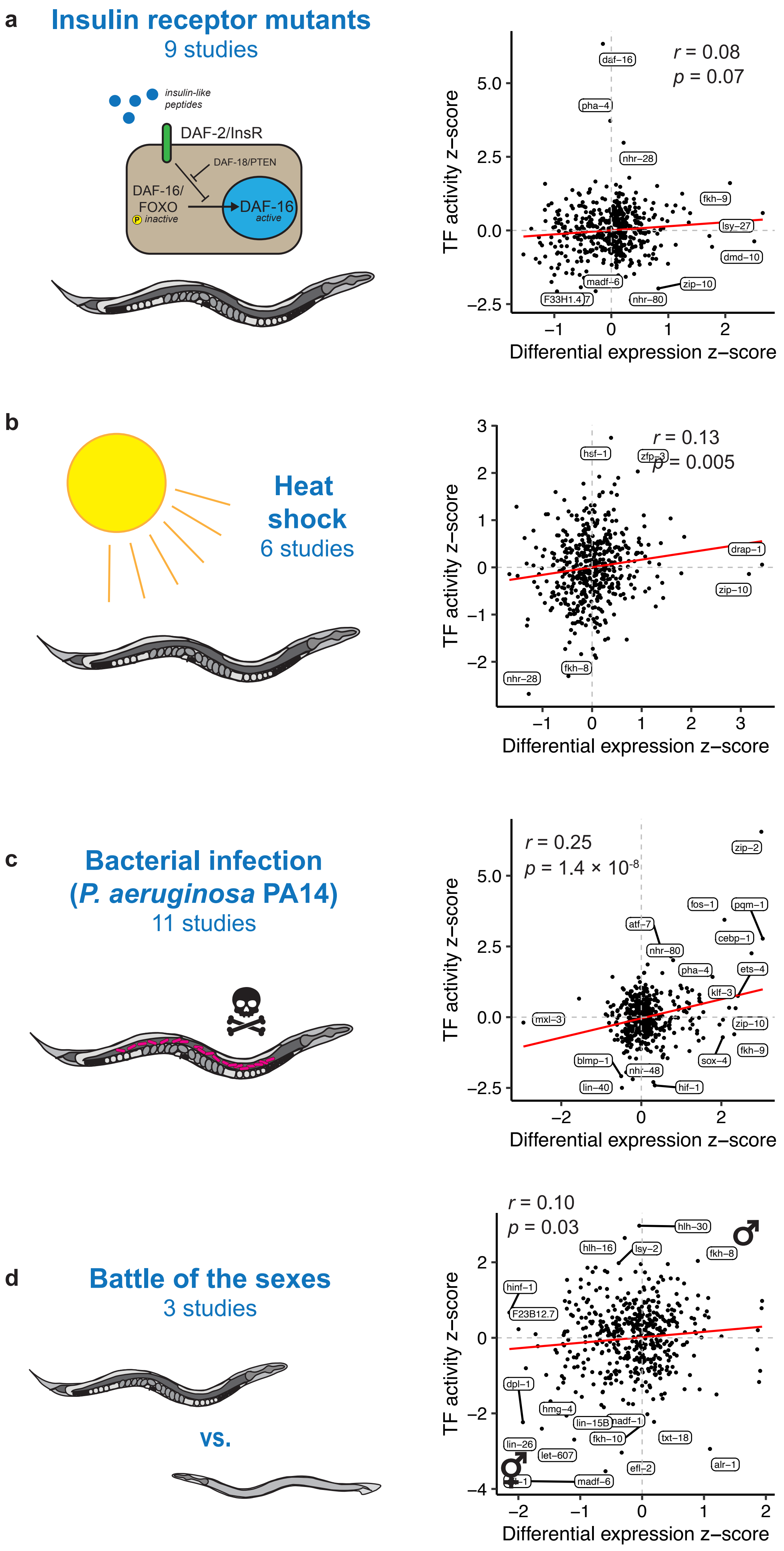

a
Insulin receptor mutants
9 studies
insulin-like peptides
DAF-2/InsR
DAF-18/PTEN
DAF-16/ FOXO
inactive
DAF-16
active
r = 0.08
p = 0.07
daf−16
pha−4
nhr−28
fkh−9
lsy−27
dmd−10
zip−10
madf−6
F33H1.4
nhr−80
TF activity z−score
Differential expression z−score
b
Heat shock
6 studies
r = 0.13
p = 0.005
hsf−1
zfp−3
drap−1
zip−10
fkh−8
nhr−28
TF activity z−score
Differential expression z−score
c
Bacterial infection
(P. aeruginosa PA14)
11 studies
r = 0.25
p = 1.4 × 10-8
zip−2
fos−1
pqm−1
atf−7
cebp−1
nhr−80
pha−4
ets−4
klf−3
mxl−3
zip−10
fkh−9
sox−4
blmp−1
nhr−48
lin−40
hif−1
TF activity z−score
Differential expression z−score
d
Battle of the sexes
3 studies
vs.
r = 0.10
p = 0.03
hlh−30
hlh−16
lsy−2
fkh−8
hinf−1
F23B12.7
dpl−1
hmg−4
lin−15B
madf−1
fkh−10
txt−18
lin−26
let−607
efl−2
alr−1
madf−6
TF activity z−score
Differential expression z−score
