# Supplementary material for: CelEst: a unified gene regulatory network for estimating transcription factor activities in C. elegans
Source: Genetics. 2024 Dec 20;229(3):iyae189. doi: 10.1093/genetics/iyae189 (PMC11912867; doi:10.1093/genetics/iyae189)
Supplement: iyae189_Supplementary_Data [file iyae189_supplementary_data.zip › Supplemental_Figure_Legends_GENETICS-2024-307499.docx]

**Fig S1. ChIP-derived GRNs perform well when potential targets in HOT regions are excluded (related to Fig 1)**

**a)** Schematic of pipeline to generate GRNs from ChIP-seq datasets.

**b)** Benchmarking performance with different TF activity estimation methods (subpanels) for GRNs derived from ChIP-seq data with no filtering of High Occupancy Target (HOT) regions. Axes show performance (AUROC/AUPRC) in benchmarking pipeline. Colours show different cut-offs for maximum number of targets per TF. ;Colours correspond to cut-offs; transparent points show mean AUROC/AUPRC of randomly shuffled networks; error bars show standard deviation. The number of experiments and unique TFs in the benchmarking set overlapping with TFs in the GRN is noted on the panel in green text.

**c)** Line shows evolution of AUPRC (above) and AUROC (below) with increasing cut-off for maximum number of targets per TF for GRNs derived from ChIP-seq data with no filtering of HOT regions. Colours indicate TF activity estimation methods. Note x-axis (cut-off) is on a log scale. Fig 1b shows benchmarking performance for AUPRC and ‘mlm’ only.

**d**) Line shows evolution of AUPRC (above) and AUROC (below) with increasing cut-off for maximum number of distinct TFs (out of 217 from Kudron *et al.* 2018) bound to a potential HOT region for overlapping target genes to be retained. Colours indicate TF activity estimation methods as in panel C. More stringent (i.e. lower) cut-offs lead to exclusion of more TFs from the GRN that retain fewer than 15 targets outside of these regions; text shows the number of TFs retained for each cut-off. No cut-off for maximum number of targets per TF was applied for these GRNs. Benchmarking set consisted of 66 / 32 (for HOT region cut-off ≤ 30) or 67 / 33 (cut-off > 30) experiments and TFs respectively.

**e)** Benchmarking performance with different TF activity estimation methods for GRNs derived from ChIP-seq data with target promoters overlapping HOT regions excluded. Details as for panel b.

**f)** Change in AUPRC (above) and AUROC (below) with increasing cut-off for ChIP-derived GRNs with HOT regions excluded for all methods. Fig 1b shows benchmarking performance for AUPRC and ‘mlm’ only.

mlm: multivariate linear model, ulm: univariate linear model, wsum: weighted sum **Fig S2. GRNs derived from promoters with best motif matches perform well with an optimal cut-off of regulon size (related to Fig 1)**

**a)** Schematic of pipeline to generate GRNs from *in vitro-*derived TF DNA binding motif datasets.

**b)** Benchmarking performance with different TF activity estimation methods (subpanels) for GRNs derived from DNA binding motifs. Axes show performance (AUROC/AUPRC) in benchmarking pipeline. Colours show different cut-offs for maximum number of targets per TF. Colours correspond to cut-offs; transparent points show mean AUROC/AUPRC of randomly shuffled networks; error bars show standard deviation. The number of experiments and unique TFs in the benchmarking set overlapping with TFs in the GRN is noted on the panel in green text. Fig 1c shows benchmarking performance for AUPRC and ‘mlm’ only.

**c)** Line shows change in AUPRC (above) and AUROC (below) with increasing cut-off for maximum number of targets per TF for GRNs derived from derived from DNA binding motif. Note x-axis (cut-off) is on a log scale.

**d)** Schematic illustrates standard pipeline (considering only the best motif match per promoter) or homotypic binding cluster pipeline (adding extra priority to promoters with multiple matches which may be imperfect).

**e)** Benchmarking performance for different cut-offs (subpanels) with targets ordered by best match or by a homotypic binding score which takes into account multiple matches per promoter. Details as for panel b.

mlm: multivariate linear model, ulm: univariate linear model, wsum: weighted sum **Fig S3. GRNs that combine targets from multiple data sources perform well despite few shared interactions (related to Fig 1)**

**a)** Benchmarking performance with different TF activity estimation methods (subpanels) for GRNs derived from TF-promoter interactions found in a large-scale enhanced yeast one-hybrid (eY1H) screen. Fig 1d shows benchmarking performance for multivariate linear model (‘mlm’) only. Transparent points show mean AUROC/AUPRC of randomly shuffled networks; error bars show standard deviation.

**b)** Benchmarking performance with different TF activity estimation methods (subpanels) for GRNs derived from ChIP-seq, DNA binding motif or a combination of the two with or without additional weight for shared targets. These GRNs are benchmarked on a common benchmarking set consisting of only TFs present in all networks to allow direct comparison. Fig 1e shows benchmarking performance for mlm only

**c)** Benchmarking performance with different TF activity estimation methods (subpanels) for the *Cel*EsT network, which covers 487 TFs by combining data from multiple experimental sources. Fig 1f shows benchmarking performance for mlm only.

**d)** Benchmarking performance with different TF activity methods (subpanels) for the combined *Cel*EsT network (with or without additional weight for interactions shared across datasets) with a benchmarking set derived from differential expression analysis comparing treatment and control groups directly without accounting for any potential difference in developmental age.

**e)** Fraction of target genes derived from ChIP-seq or promoter motifs which are found in stable ‘active’ or ‘regulated’ chromatin domains, as defined in Evans *et al.* 2016. p-values from chi-squared test.

**f)** Violin plot of Gini coefficient for gene expression across tissues for TF targets derived from ChIP-seq or promoter motifs. p-values are from Kruskal Wallis/Dunn’s post-hoc tests. Gini coefficient for each gene was calculated using tissue-specific gene expression for L2 larvae reported by Cao *et al.* 2017. A Gini coefficient of 1 indicates highly unequal expression, whereas a low Gini coefficient indicates equal expression across tissues.

**g)** Enrichments for tissue-specific genes (reported by Cao *et al.* 2017) among target genes derived from ChIP-seq or promoter motifs. The number of tissue-specific genes for each tissue is indicated in parentheses after the tissue type on the x axis. Error bars show 95% confidence intervals. Bubble size indicates p-value (Fisher’s exact test).

NS, not significant.

**Fig S4. *Cel*EsT performance is not driven by a small number of high-quality TFs**

**a, b)** Benchmarking performance for TFs from major families with (**a**) or without (**b**) correction for inferred developmental age. The point size reflects the number of benchmarking experiments; the size of the interior ring reflects the number of unique TFs. Faded points and error bars reflect mean/SD of AUROC/AUPRC for each family for 100 random shuffled networks.

**c, d**) Benchmarking performance for individual TFs with (**c**) or without (**d**) correction for inferred developmental age. Point colour reflects TF family; point size reflects number of benchmarking experiments. The best/worst TFs are labelled individually. Points labelled in **c** are also labelled in **d** and vice versa.

**e, f**) Benchmarking performance with (**e**) or without (**f**) correction for inferred developmental age for the *Cel*EsT network (red dot) and upon removal of 10 TFs at a time from the benchmarking set (~25% of the total). Each grey dot represents one of 1000 trials removing a subset of TFs.

Legend below show colours for families, as well as benchmarking experiments, unique TFs and total TFs for each.

**Fig S5. Motif conservation-based filtering of target genes to create GRNs with equal performance with fewer TF-target interactions (related to Fig 2)**

**A)** Heatmap shows benchmarking pipeline performance (AUROC left, AUPRC right) for motif-based GRNs with different initial elegans cut-offs and different cross-species conservation probability FDR cut-offs. The box shows the chosen network (top 1500 *elegans* targets filtered by FDR of 0.5) with the best performance for both AUROC and AUPRC.

**b)** Benchmarking performance with different TF activity estimation methods (subpanels) for motif-based GRNs after conservation-based filtering (red) versus the best-performing *elegans*-only motif-based GRN (‘full 1000’, purple), a control network with the same number of targets per TF selected from the best *elegans* motif matches (‘control’, grey) and the *Cel*EsT network subsetted to the same TFs (‘CelEsT*’, green). Axes show performance (AUROC/AUPRC) in benchmarking pipeline. Transparent points show mean AUROC/AUPRC of randomly shuffled networks; error bars show standard deviation. The number of experiments and unique TFs in the benchmarking set overlapping with TFs in the GRN is noted on the panel in green text. Fig 2b shows benchmarking performance for ‘mlm’ only.

**c)** Line shows change in AUPRC (above) and AUROC (below) with increasing cut-off for maximum number of targets per TF for GRNs derived from ChIP-seq data with (right) or without (left) exclusion of target genes within HOT regions. Colours indicate network with targets ordered by conservation probability from ChIP-derived *de novo* motifs (red), known motifs from CisBP where applicable, else *de novo* motifs (pruple) or ChIP peak signal strength (grey). Note x-axis (cut-off) is on a log scale. Experiment/unique TF numbers in green text. Related to Fig 2d.

**d)** Benchmarking performance with different TF activity estimation methods for the orth*Cel*EsT network. Details as for panel b. Fig 2e shows benchmarking performance for ‘mlm’ only.

**e)** Benchmarking performance with different TF activity estimation methods for the max*Cel*EsT network. Details as for panel b.

**Fig S6. *Cel*EsT shows TF activity changes in mutants of multiple insulin/IGF-1-like signalling pathway components (related to Fig 3).**

Volcano plots shows mean TF activity z-score and geometric mean *p*-value. The bubble size is proportional to the *p*-value.

**a)** 9 studies (see Fig S7a and Table S6) comparing the severe *daf-2(e1370)* allele to wildtype controls. Note this panel is identical to that shown in Fig 3a and is provided here for ease of comparison.

**b)** 2 studies (see Fig S7b and Table S6) comparing *daf-2(e1370); daf-16(mu86)* to *daf-2(e1370)*. Note that one of these two studies was included in the benchmarking set used to evaluate GRN performance.

**c)** 1 study (Table S6) comparing *daf-2(e1370); daf-18(ok480)* to *daf-2(e1370)*.

**d)** 3 studies (see Fig S7b and Table S6) comparing *daf-16* null alleles to wildtype controls.

**Fig S7. TF activity heatmaps for analyses combining results from multiple studies (related to Fig 3).**

Heatmaps show TF activity z-scores for each study considered for analysis for each condition. Study characteristics are annotated below; dendrograms above show study hierarchical clustering. Studies which were excluded as outliers and not included in the analyses shown in Fig 3 or Fig S6 are shaded out and marked with red text.

**a)** 10 studies (1 excluded; Table S6) comparing a severe mutant allele of the insulin receptor orthologue *daf-2* to wildtype controls. Related to Fig 3b, Fig S6a and Fig S8a.

**b)** 2 studies (Table S6) comparing *daf-2(e1370); daf-16(mu86)* to *daf-2(e1370)*. Related to Fig S6b.

**c)** 5 studies (2 excluded; Table S6) comparing *daf-16(mu86)* animals to wildtype controls. Related to Fig S6d.

**d)** 7 studies (1 excluded; Table S6) comparing heat-shock treated animals to untreated controls. Related to Fig 3c and Fig S8b.

**e)** 11 studies (Table S6) comparing animals infected with the pathogenic bacterium *P. aeruginosa* strain PA14 to unexposed controls. Related to Fig 3d and Fig S8c.

**f)** 4 studies (1 excluded; Table S6) comparing transcriptomes of male animals to hermaphrodites. Related to Fig 3e and Fig S8d.

**Fig S8. TF activity is independent of differential expression in many conditions but strongly related in bacterial infection (related to Fig 3).**

Scatterplots show mean differential expression z-scores for TF-encoding genes on the x-axis and *Cel*EsT-inferred activity for those TFs. Each plot is annotated with Pearson’s correlation coefficient (*r*) and *p*-value.

All analyses controlled for developmental age during differential expression analysis, with DE stats then analysed using the multivariate linear model TF activity estimation method and the *Cel*EsT network.

**a)** Analysis of 9 studies (Table S6) comparing a severe mutant allele of the insulin receptor orthologue *daf-2* to wildtype controls. See also Fig 3b and Fig S7a.

**b)** Analysis of 6 studies (Table S6) comparing heat-shocked animals to untreated control animals. See also Fig 3c and S7d.

**c)** Analysis of 11 studies (Table S6) comparing animals exposed to the pathogenic bacterium *Pseudomonas aeruginosa* PA14 to untreated controls. See also Fig 3d and S7e.

**d)** Analysis of 3 studies (Table S6) comparing the transcriptome of male animals to that of hermaphrodites. See also Fig 3e and S7f.**Fig S9. Co-expression derived gene regulatory network inferred by SJARACNe from a large RNA-seq dataset has no predictive capacity in benchmarking pipeline.**

TF activity estimation benchmarking performance for various methods for a GRN based on co-expression using edges computed by the SJARACNe algorithm using a large RNA-seq dataset from the CeNDR resource.
