# Supplementary figures and images for: CelEst: a unified gene regulatory network for estimating transcription factor activities in C. elegans
Source: Genetics. 2024 Dec 20;229(3):iyae189. doi: 10.1093/genetics/iyae189 (PMC11912867; doi:10.1093/genetics/iyae189)

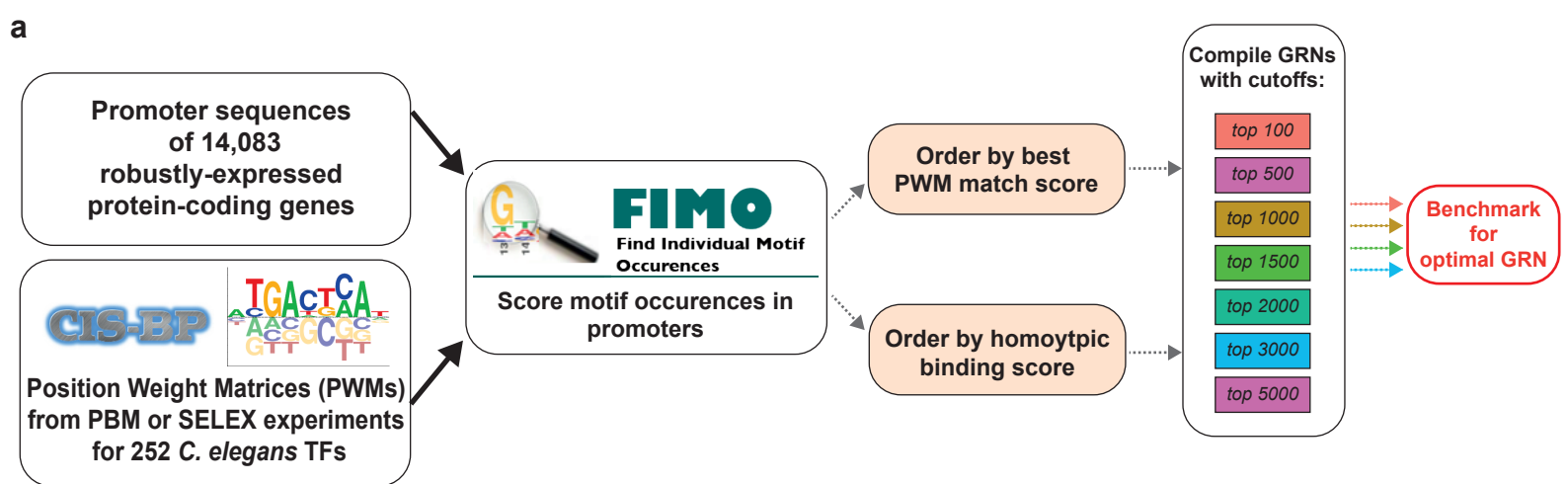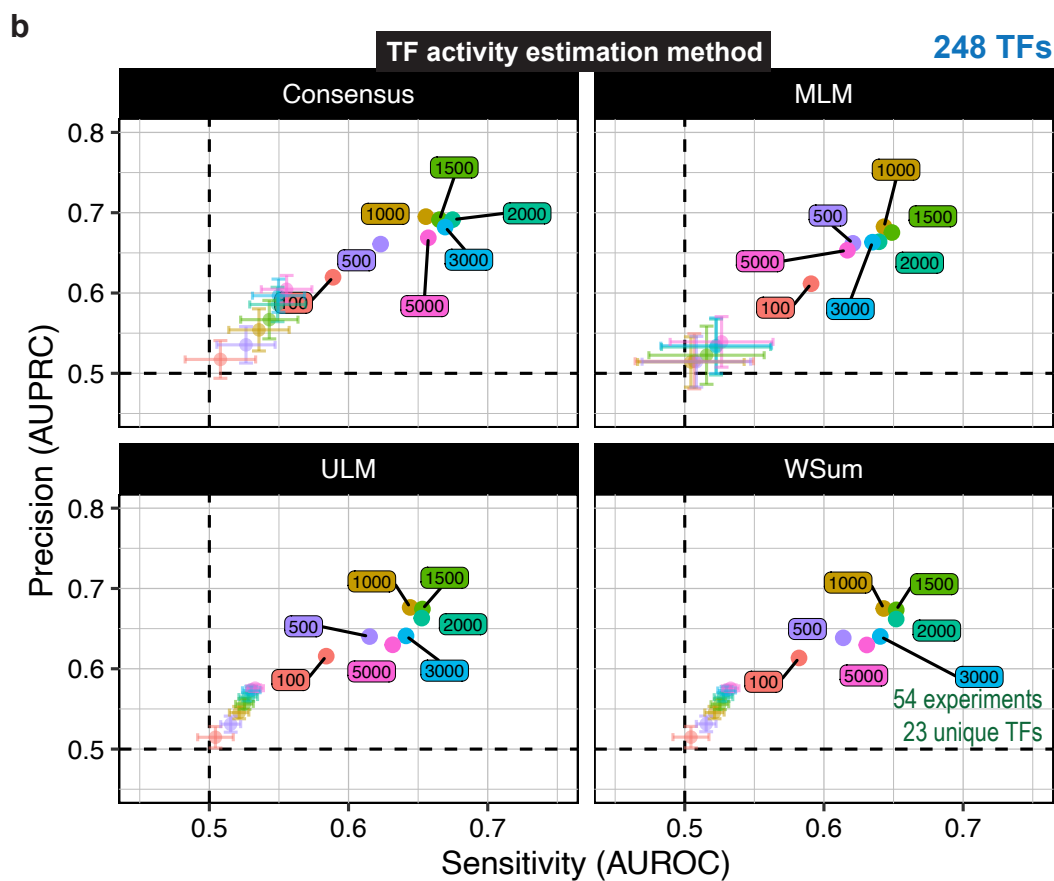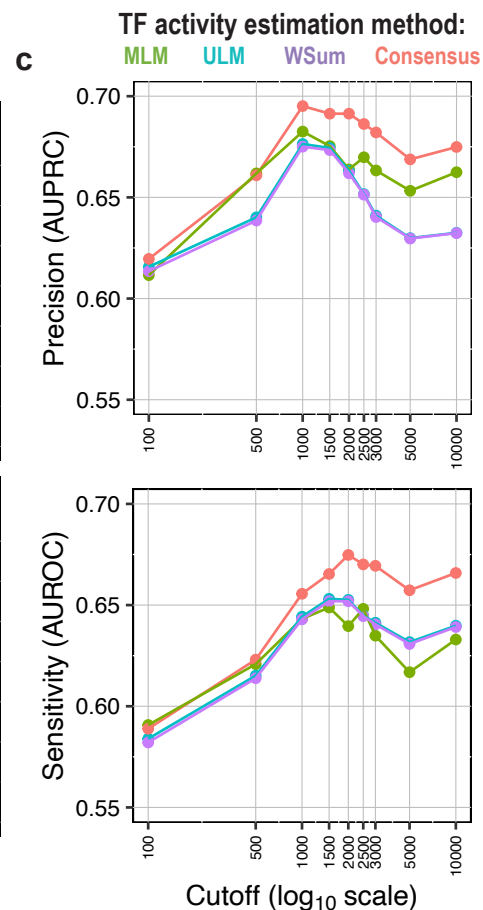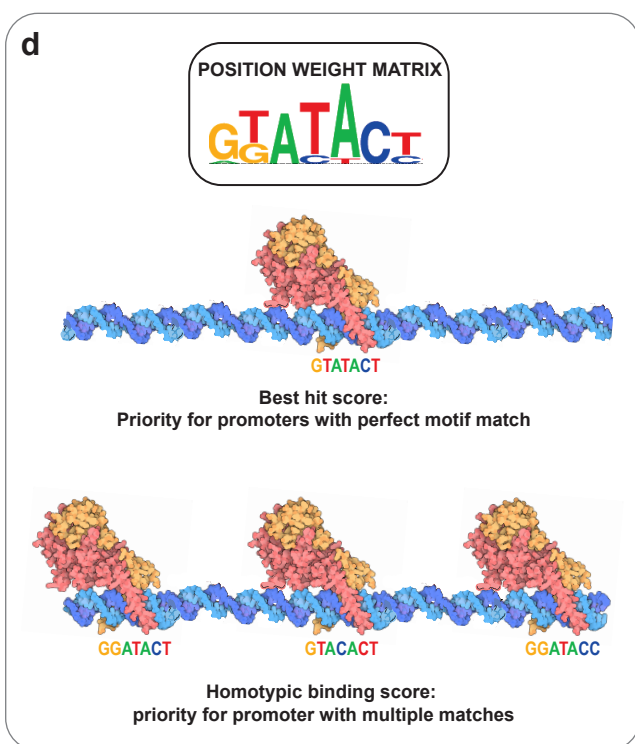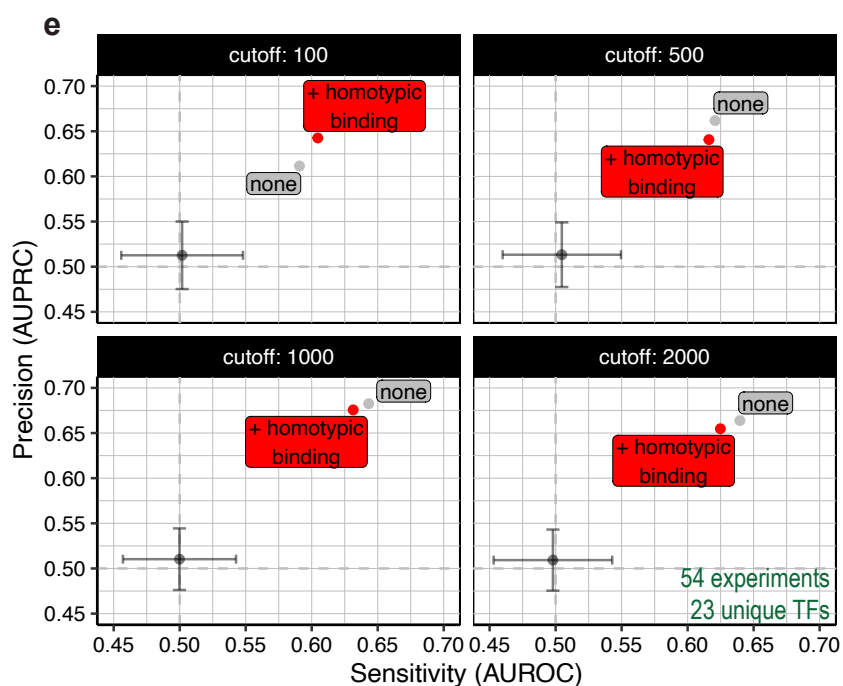

Supplement: iyae189_Supplementary_Data [file iyae189_supplementary_data.zip › Figure_S2_GENETICS-2024-307499.pdf]

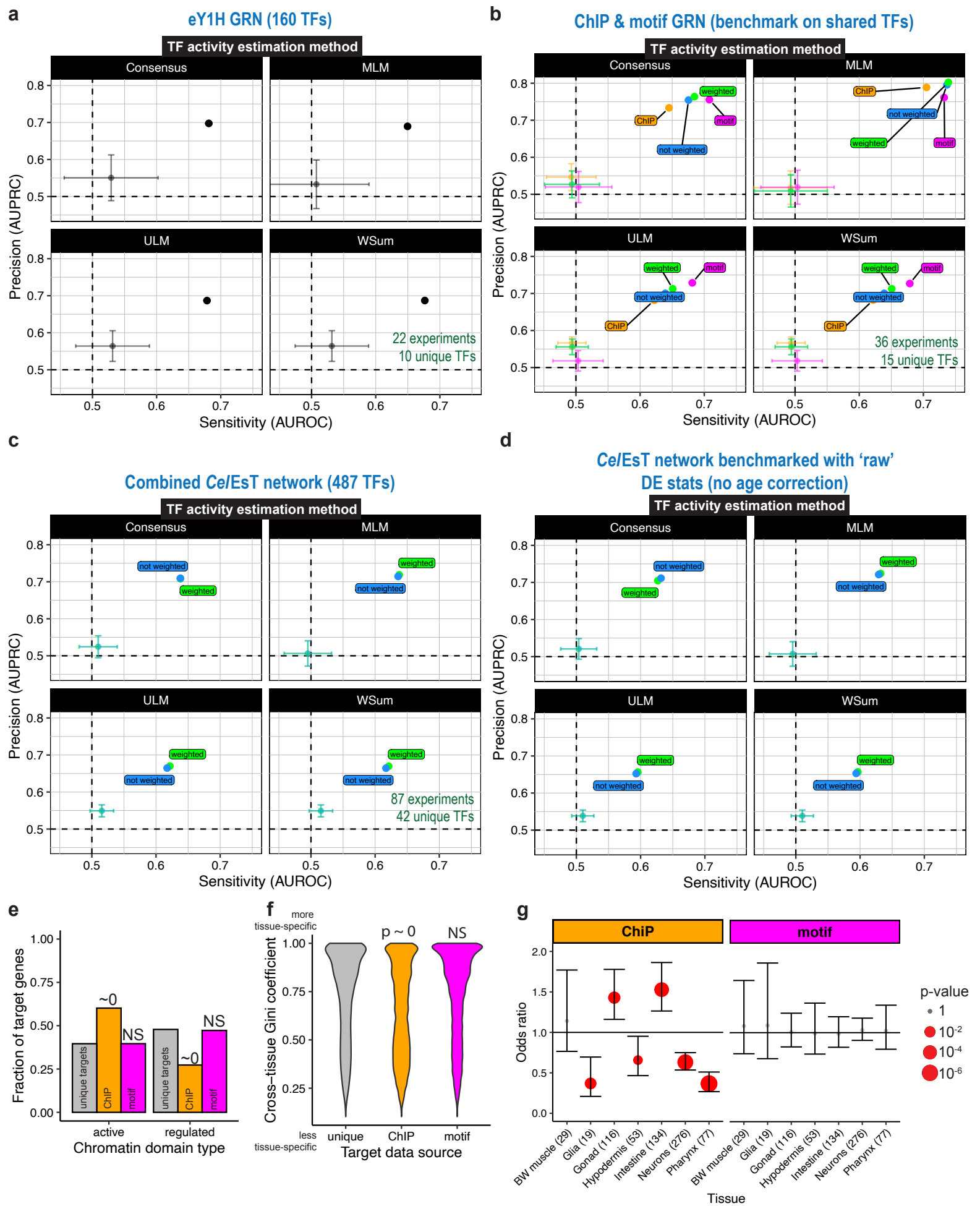

Supplement: iyae189_Supplementary_Data [file iyae189_supplementary_data.zip › Figure_S3_GENETICS-2024-307499.pdf]

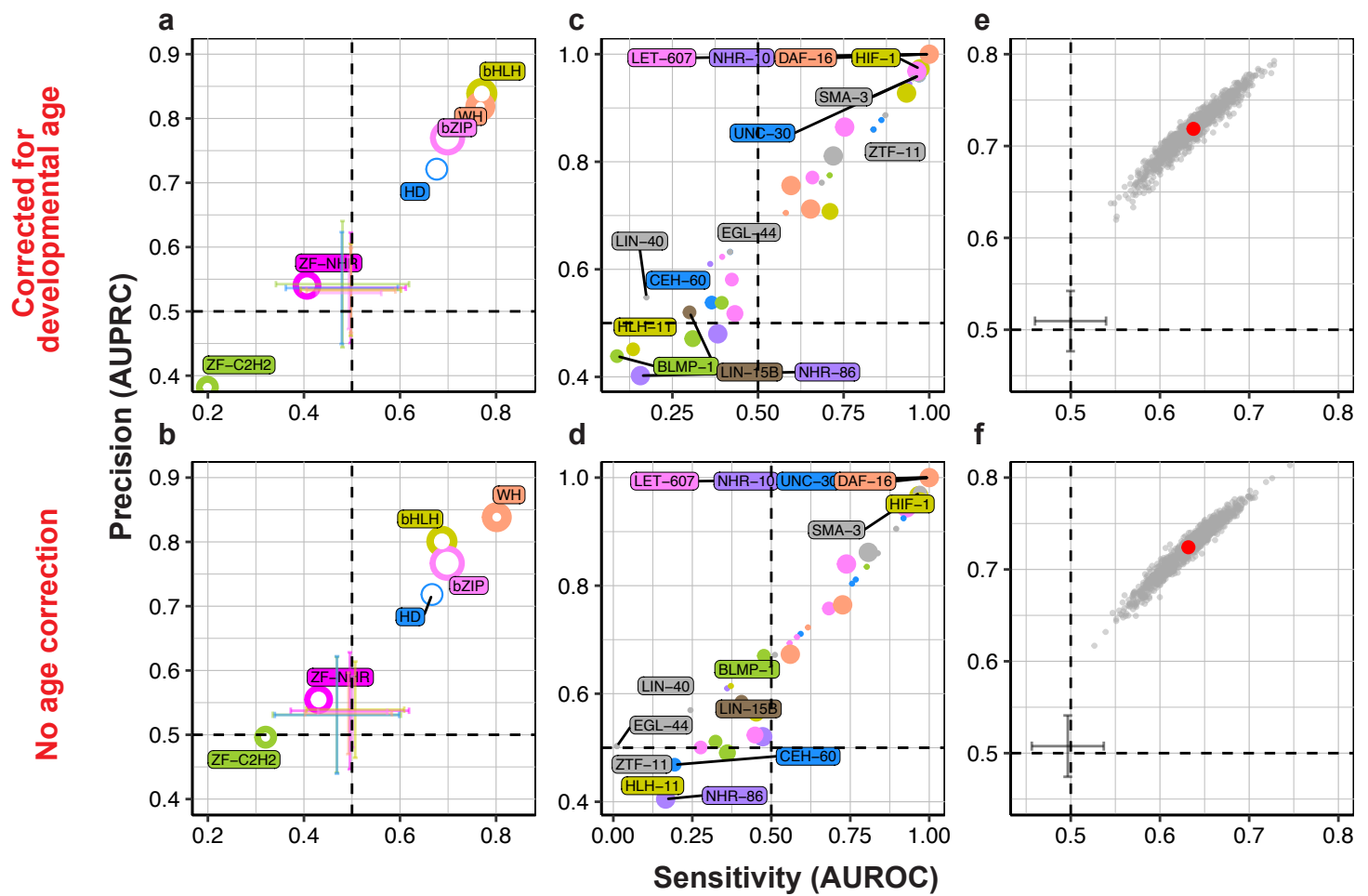

Supplement: iyae189_Supplementary_Data [file iyae189_supplementary_data.zip › Figure_S4_GENETICS-2024-307499.pdf]

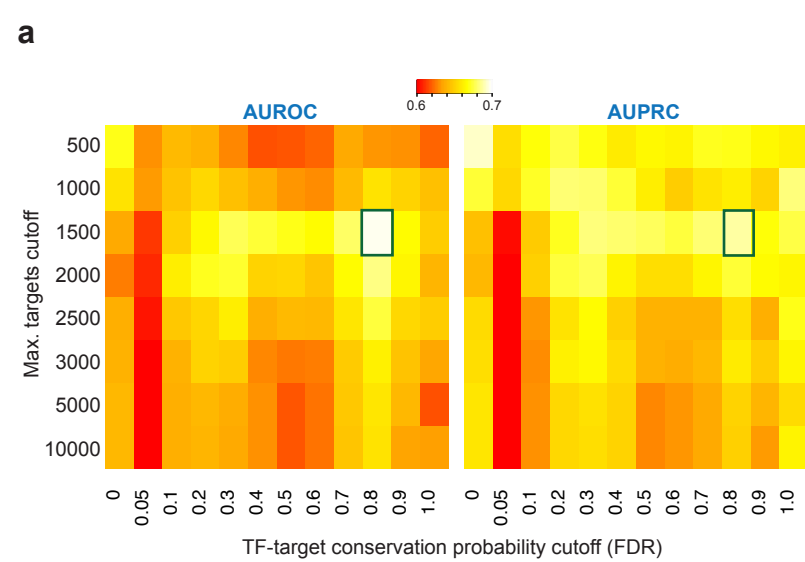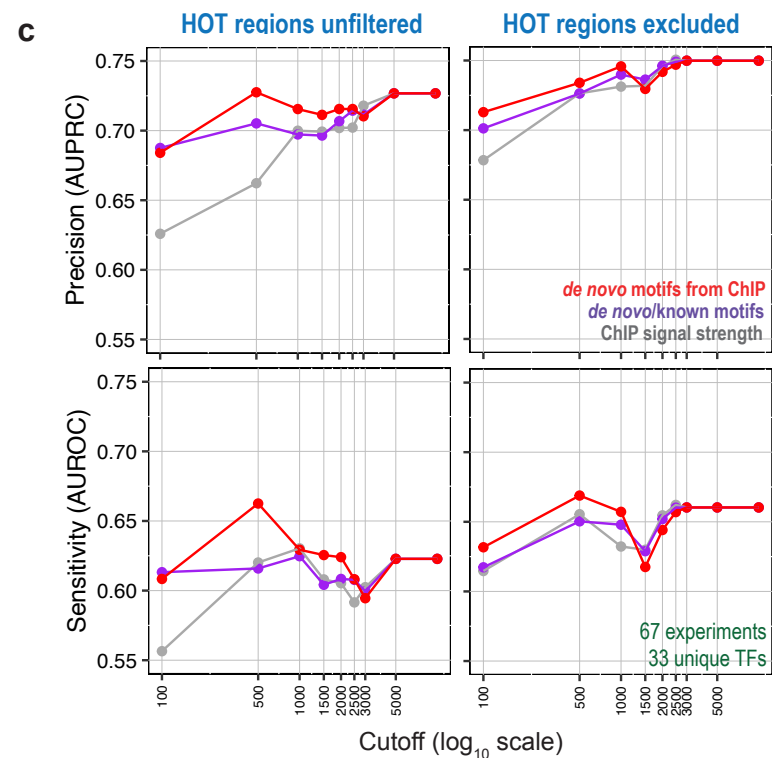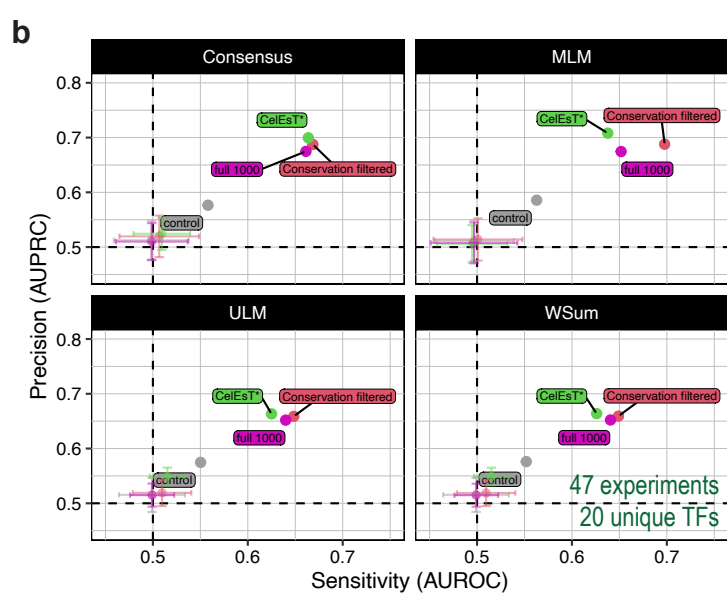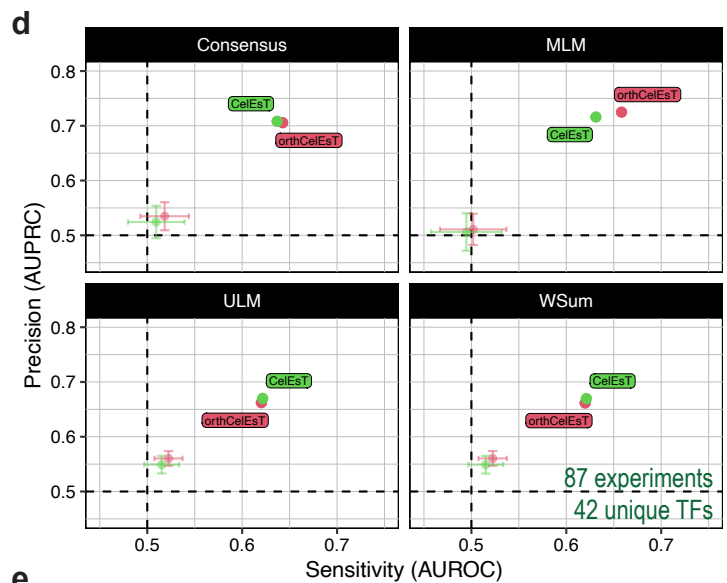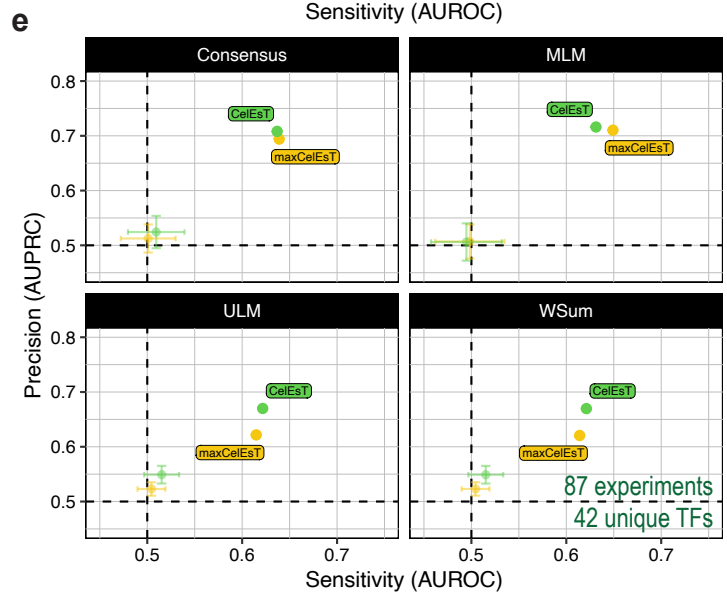

Supplement: iyae189_Supplementary_Data [file iyae189_supplementary_data.zip › Figure_S5_GENETICS-2024-307499.pdf]

## Insulin-like signalling mutants

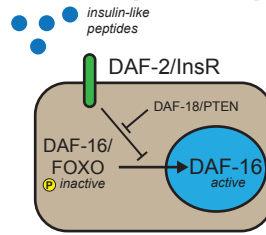

**a** *daf-2(e1370)*

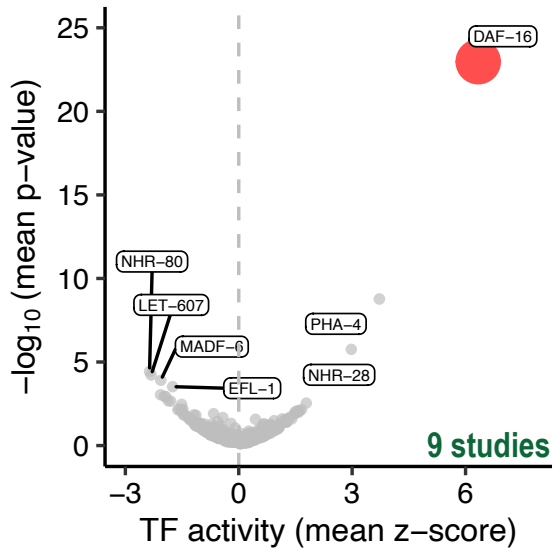

**b** *daf-2(e1370); daf-16(null)*

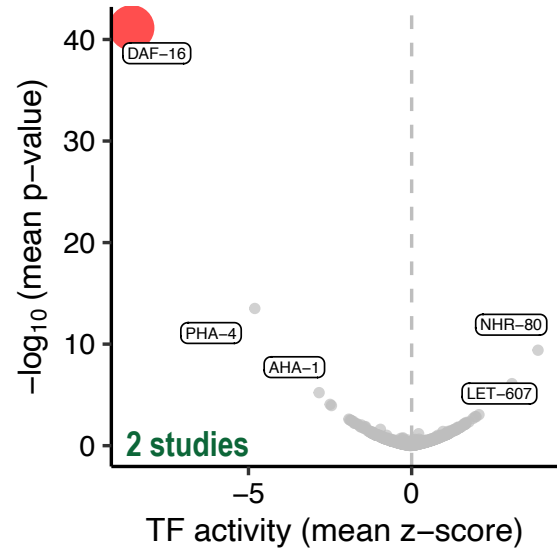

**c** *daf-2(e1370); daf-18(null)*

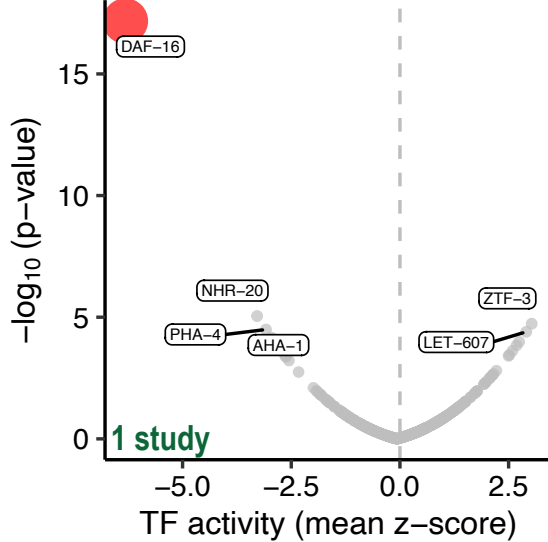

**d** *daf-16(null)*

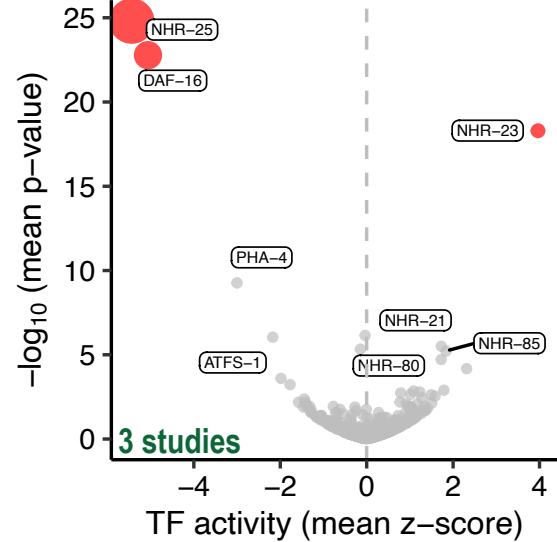

Supplement: iyae189_Supplementary_Data [file iyae189_supplementary_data.zip › Figure_S6_GENETICS-2024-307499.pdf]

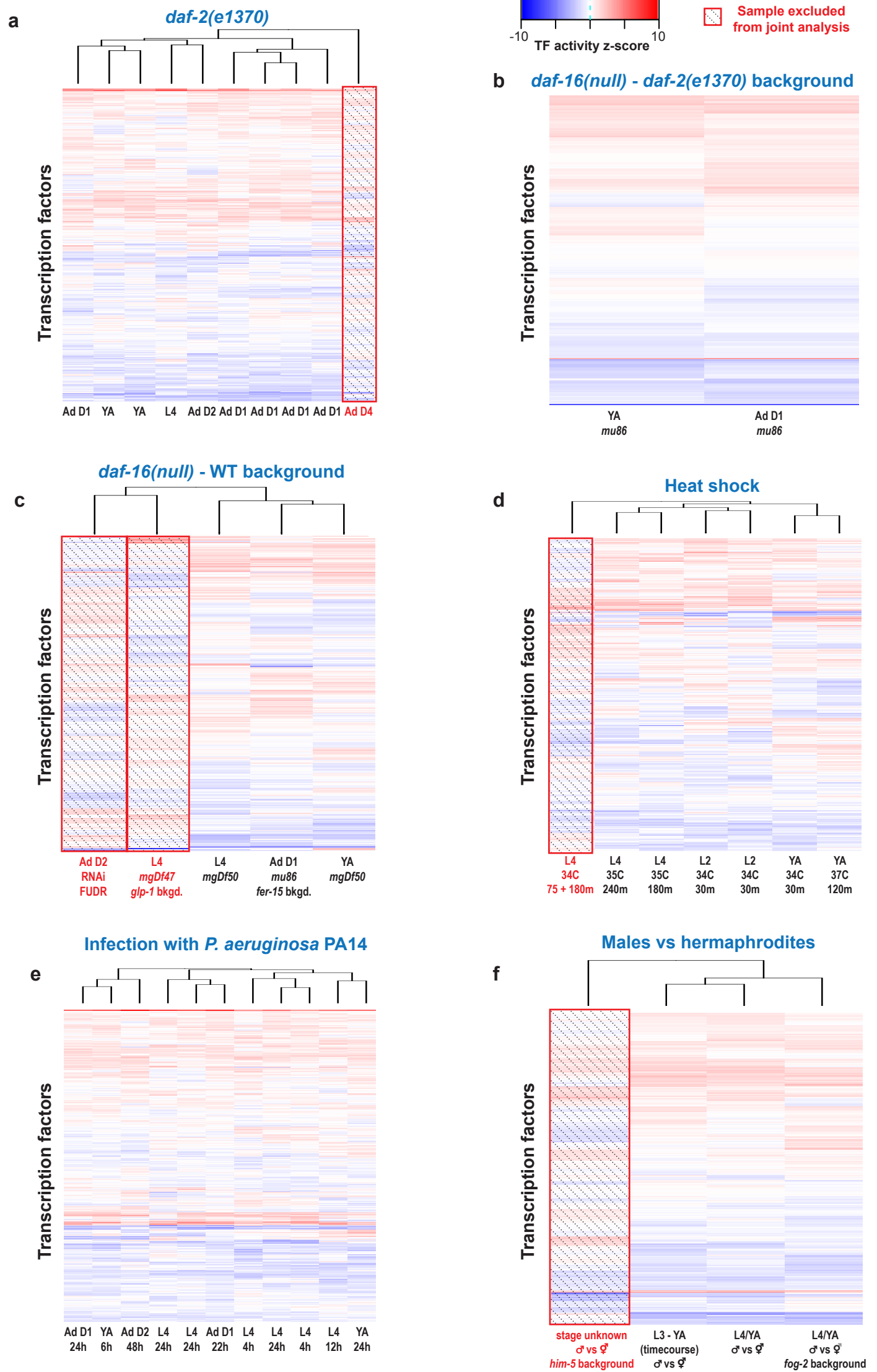

Supplement: iyae189_Supplementary_Data [file iyae189_supplementary_data.zip › Figure_S7_GENETICS-2024-307499.pdf]

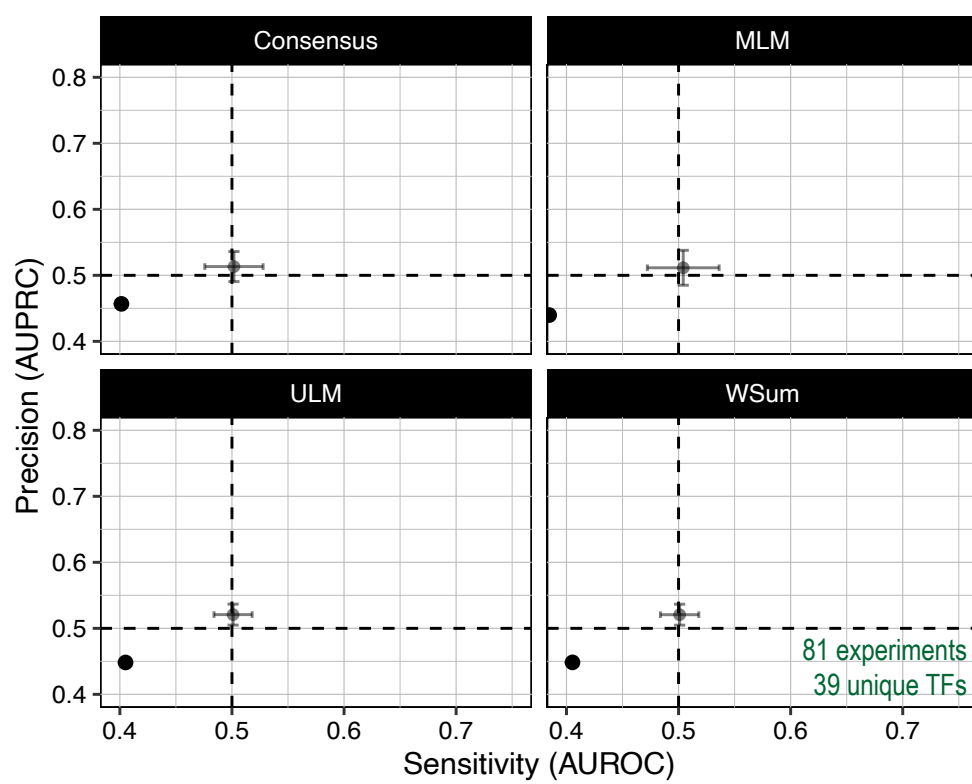

Supplement: iyae189_Supplementary_Data [file iyae189_supplementary_data.zip › Figure_S9_GENETICS-2024-307499.pdf]
